# Experimental evaluation of the zoonotic infection potency of simian retrovirus type 4 using humanized mouse model

**DOI:** 10.1038/srep14040

**Published:** 2015-09-14

**Authors:** Kei Sato, Tomoko Kobayashi, Naoko Misawa, Rokusuke Yoshikawa, Junko S. Takeuchi, Tomoyuki Miura, Munehiro Okamoto, Jun-ichirou Yasunaga, Masao Matsuoka, Mamoru Ito, Takayuki Miyazawa, Yoshio Koyanagi

**Affiliations:** 1 Laboratory of Viral Pathogenesis, Institute for Virus Research, Kyoto University, Kyoto, Japan.; 2 Laboratory of Signal Transduction, Institute for Virus Research, Kyoto University, Kyoto, Japan.; 3 Laboratory of Virolution, Institute for Virus Research, Kyoto University, Kyoto, Japan.; 4 Laboratory of Primate Model, Institute for Virus Research, Kyoto University, Kyoto, Japan.; 5 Laboratory of Virus Control, Institute for Virus Research, Kyoto University, Kyoto, Japan; 6 Center for Human Evolution Modeling Research, Primate Research Institute, Kyoto University, Inuyama, Aichi, Japan; 7 Central Institute for Experimental Animals, Kawasaki, Kanagawa, Japan; 8 CREST, Japan Science and Technology Agency, Saitama, Japan

## Abstract

During 2001-2002 and 2008-2011, two epidemic outbreaks of infectious hemorrhagic disease have been found in Japanese macaques (*Macaca fuscata*) in Kyoto University Primate Research Institute, Japan. Following investigations revealed that the causative agent was simian retrovirus type 4 (SRV-4). SRV-4 was isolated by using human cell lines, which indicates that human cells are potently susceptible to SRV-4 infection. These raise a possibility of zoonotic infection of pathogenic SRV-4 from Japanese macaques into humans. To explore the possibility of zoonotic infection of SRV-4 to humans, here we use a human hematopoietic stem cell-transplanted humanized mouse model. Eight out of the twelve SRV-4-inoculated humanized mice were infected with SRV-4. Importantly, 3 out of the 8 infected mice exhibited anemia and hemophagocytosis, and an infected mouse died. To address the possibility that SRV-4 adapts humanized mouse and acquires higher pathogenicity, the virus was isolated from an infected mice exhibited severe anemia was further inoculated into another 6 humanized mice. However, no infected mice exhibited any illness. Taken together, our findings demonstrate that the zoonotic SRV-4 infection from Japanese macaques to humans is technically possible under experimental condition. However, such zoonotic infection may not occur in the real society.

Simian type D retroviruses, a lineage of the genus *Betaretrovirus*, are enzootic in Asian monkeys (the genus *Macaca*) such as cynomolgus monkeys (*Macaca fascicularis*) and rhesus macaques (*Macaca mulatta*)^1^,^2^,^3^. Although mild illness such as anemia and diarrhea can be occasionally observed in infected cynomolgus monkeys, the infections of simian type D retroviruses are usually benign and asymptomatic in these monkeys^3^,^4^.

During 2001-2002 and 2008–2011, in Kyoto University Primate Research Institute (KUPRI), Japan, Japanese macaques (*Macaca fuscata*), a species of the genus *Macaca*, died of a hemorrhagic syndrome with profound symptoms such as anorexia, pallor, and severe anemia with thrombocytopenia^5^,^6^,^7^. Extensive laboratory investigations revealed that the causative agent was a simian type D retrovirus, simian retrovirus type 4 (SRV-4)^5^,^6^,^7^,^8^. Although SRV-4 infection is usually benign in cynomolgus monkeys^9^, SRV-4 infection in Japanese macaques has not been reported so far. Therefore, the reason why and how SRV-4 infection leads to high pathogenicity and mortality in Japanese macaques had not been reported until we recently reported^6^,^7^. By experimental infection of Japanese macaques with an SRV-4 isolate and molecularly cloned SRV-4, we reproduced severe thrombocytopenia in all six SRV-4 inoculated Japanese macaques within 31 days after virus inoculation^6^. We detected SRV-4 proviruses in blood cells and SRV-4 RNA in plasma from 3 to 11 days post infection. The viral RNA copy numbers in plasma stayed at a high level (10^5^ to 10^7^ copies/ml) until the onset of severe thrombocytopenia^6^. We also demonstrated that the receptor for SRV-4 in Japanese macaque is ASCT2, a neutral amino acid transporter^6^. *ASCT2* mRNA is expressed in a variety of tissues and could be detected at a high level in stomach, colon, lung, thymus, spleen, lymph nodes, testis, and prostate^6^. In parallel with ASCT2 expression levels, the SRV-4 proviral copy numbers in digestive and lymph organs were relatively high in SRV-4-infected Japanese macaques, reaching to 0.005 to 0.04 copies per cell (relative copy numbers to *GAPDH* gene)^6^. Intriguingly, we could not detect any anti-SRV-4 antibodies in almost all Japanese macaques showing severe thrombocytopenia^6^,^7^, which may indicate that the majority of Japanese macaques are immunologically tolerant to SRV-4. The pathobiological mechanism of the diseases induced by SRV-4 in Japanese macaques is still unknown at present. Therefore, the reason why and how SRV-4 infection leads to high pathogenicity and mortality in Japanese macaques in KUPRI remains unveiled.

So far, Lerche *et al*. have reported that the humans, who occupationally exposed to nonhuman primates such as the genus *Macaca*, can be infected with simian type D retroviruses^10^. Although certain reports have suggested that betaretroviruses can infect humans and are associated with some illness such as breast cancer with primary biliary cirrhosis^11^,^12^,^13^,^14^, this concept is still controversial^15^,^16^,^17^, and there is no generally accepted consensus that retrovirus infection causes disease in humans except for human T-cell leukemia virus (HTLV) and human immunodeficiency virus (HIV) infections. However, it is of particular importance that human cell lines such as 293T cells (human embryonic kidney cell line), Raji cells (human B cell line), and TE671 cells (human rhabdomyosarcoma cell line)^18^ were used for the SRV-4 isolation from infected Japanese macaques with severe symptoms^6^. Since human cells are intrinsically susceptible to SRV-4 infection, we cannot exclude the possibility of zoonotic transmission of pathogenic SRV-4 into human population.

In order to reproduce and elucidate the infections of human-specific pathogens such as HIV *in vivo*, we have constructed a humanized mouse model by xenotransplanting human CD34^+^ hematopoietic stem cells (HSCs) into an immunodeficient NOD/SCID *Il2rg*^–/–^ (NOG) mouse^19^,^20^,^21^,^22^,^23^,^24^,^25^. In this system, human leukocytes are differentiated *de novo* and are stably and longitudinally maintained. By using these humanized mice, we have established novel animal models for HIV and Epstein-Barr virus (EBV) infections and related diseases^19^,^20^,^21^,^22^,^23^,^24^,^25^,^26^. It is particularly noteworthy that our humanized mice, named NOG-hCD34 mice, are able to recapitulate the pathogenesis of EBV infection including hemophagocytosis and normocytic anemia with high mortality^23^.

Recently, it has been revealed that SRV-4 preferentially replicates in the lymphoid tissues such as spleen, bone marrow (BM), lymph nodes, and tonsil of the Japanese macaques infected with SRV-4^6^. Since our humanized mice have the ability to maintain human leukopoiesis, we can test the possibility of SRV-4 infection in human by using humanized mouse model. Here, by utilizing our humanized mouse model, we demonstrate that the SRV-4 isolated from Japanese macaques with severe illness in KUPRI is able to cause zoonotic transmission into humans and potently exhibit pathogenic effects. However, our results also suggest that such zoonotic transmission may not naturally occur in the real society.

## Results

### SRV-4 inoculation into humanized mouse model

**Figure 1:**
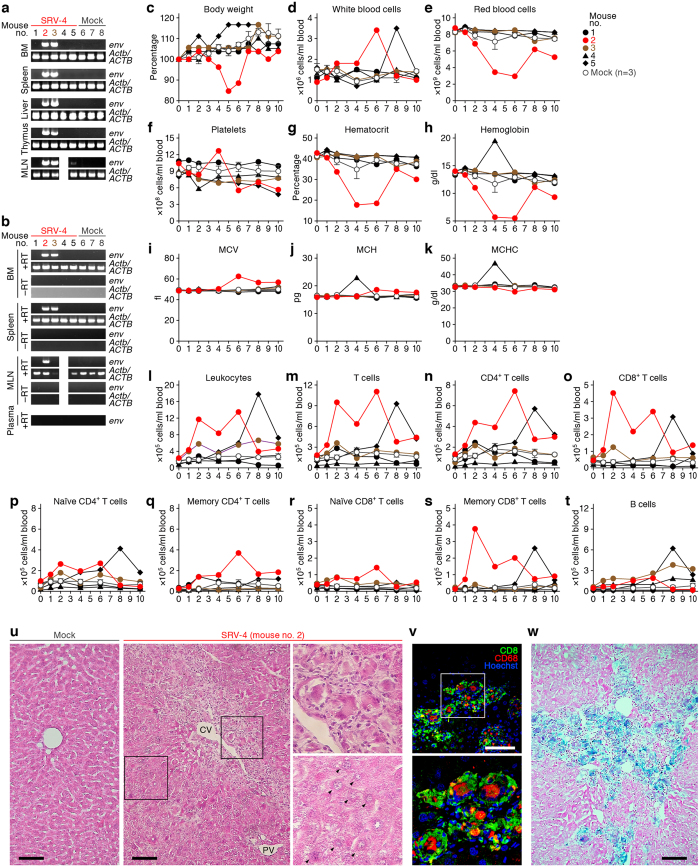
Dynamics of SRV-4 infection in humanized mouse model (experiment 1). (**a,b**) Proviral DNA (**a**) and viral RNA (**b**) were respectively analyzed by PCR and RT-PCR (targeted to *env* region). *ACTB* and *Actb* (the murine ortholog of human *ACTB*) were used as internal control, and the RT-PCR without reverse transcriptase (–RT) was used as negative control. In a and b, the results of MLN in mouse no. 4 are not shown because MLN was not detected. (**c-t**) Longitudinal analyses on the dynamics of SRV-4 infection. X-axes indicate wpi. (**c**) The body weights were routinely measured and were shown as the ratio to the initial weight. (**d-k**) The numbers of white blood cells (**d**) red blood cells (**e**) and platelets (**f**) hematocrit (**g**) hemoglobin concentration (**h**) and the values of MCV (**i**), MCH **(j**) and MCHC (**k**) were measured by hematometry. (**l-t**) The numbers of human CD45^+^ leukocytes (**l**), CD3^+^ T cells (**m**) CD4^+^ T cells (CD3^+^CD4^+^ cells; (**n**) CD8^+^ T cells (CD3^+^CD8^+^ cells; (**o**) naïve CD4^+^ T cells (CD3^+^CD4^+^CD45RA^+^ cells; (**p**) memory CD4^+^ T cells (CD3^+^CD4^+^CD45RA^–^ cells; (**q**) naïve CD8^+^ T cells (CD3^+^CD8^+^CD45RA^+^ cells; (**r**) memory CD8^+^ T cells (CD3^+^CD8^+^CD45RA^–^ cells; (**s**) and CD19^+^ B cells (**t**) were analyzed by flow cytometry and hematometry. In c-t, red, an SRV-4-infected mouse exhibited anemia (no. 2); brown, an SRV-4-infected mouse (i.e., viral DNA was detected; no. 3); and black, SRV-inoculated mice without viral replication. The data from mock-infected mice (n = 3) is presented as averages with SEMs. The mouse numbers correspond to those in Table 1. (u-w) Hemophagocytosis in the liver of an SRV-4-infected mice exhibited anemia (no. 2). The liver section of the mouse was assessed by H&E staining (u, right), immunostaining (**v**), and Berlin blue staining (**w**), and the representatives are shown. In u, H&E staining of the liver section of mock-infected mouse is presented on the left panel. In u and v, areas enclosed with squares are enlarged in independent panels. Arrowheads indicate the degenerated hepatocytes. In w, the cells stained with blue indicate hemosiderin-containing hemophagocytic macrophages. CV, central vein; PV, portal vein. Scale bar, 100 μm. In a and b, gels have been cropped; full uncropped gels are available as Supplementary Figure 1.

**Figure 2:**
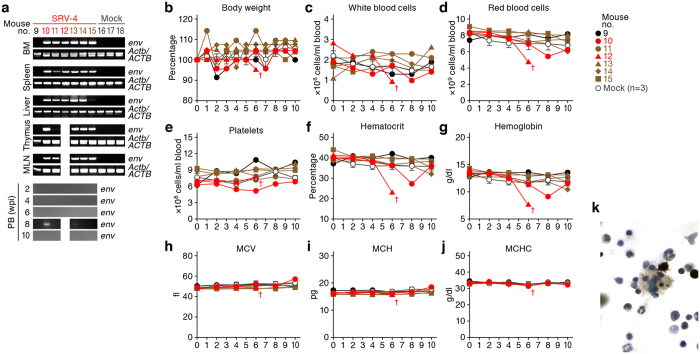
Dynamics of SRV-4 infection in humanized mouse model (experiment 2). (**a**) Proviral DNA was analyzed by PCR (targeted to *env* region). *Actb*/*ACTB* were used as internal control. In a, the results of thymus, MLN, and PB at 8 and 10 wpi in mouse no. 12 are not shown because this mouse was suddenly died at 6 wpi. (**b-j**) Longitudinal analyses on the dynamics of SRV-4 infection. X-axes indicate wpi. (**b**) The body weights were routinely measured and were shown as the ratio to the initial weight. (**c-j**) The numbers of white blood cells (**c**), red blood cells (**d**), platelets (**e**), hematocrit (**f**), hemoglobin concentration (**g**), and the values of MCV (**h**), MCH (**i**), and MCHC (**j**) were measured by hematometry. In b-j, red, SRV-4-infected mice exhibited anemia (no. 10 and 12); brown, SRV-4-infected mouse (i.e., viral DNA was detected; no. 11, 13-15); and black, an SRV-inoculated mouse without viral replication. Note that mouse no. 12 exhibited anemia and suddenly died at 6 wpi, which is indicated by daggers in b-j. The data from mock-infected mice (n = 3) were presented as the averages with SEMs. The mouse numbers correspond to those in Table 1. (k) Hemophagocytosis in the BM of an SRV-4-infected mice exhibited anemia (no. 10). The BM fluid smear was assessed by NSE staining, and a representative result is shown. Macrophages (brown) and neutrophils (blue) are stained. In a, gels have been cropped; full uncropped gels are available as Supplementary Figure 2.

In order to investigate the impact of SRV-4 infection *in vivo*, SRV-4, which is isolated from a Japanese macaque with severe thrombocytopenia^5^,^6^,^7^, was intraperitoneally inoculated into humanized mice. We performed two independent experiments, and in total, 19 humanized mice, which were reconstituted with human CD34^+^ hematopoietic stem cells from 3 individual donors, were used for this study (Table 1). For the experiment 1 (Fig. 1), 5 mice were inoculated with SRV−4 (4.8 × 10^5^ 50% tissue culture infectious dose [TCID_50_] per mouse) and 3 mice were used for mock infection. For the experiment 2 (Fig. 2), 7 mice were inoculated with SRV−4 (8.4 × 10^5^ TCID_50_ per mouse) and 3 mice were used for mock infection.

As shown in Figs 1a and 2a, SRV-4 proviral DNA was detected in the BM, spleen, liver, thymus, and mesenteric lymph node (MLN) of 8 out of the 13 SRV-4-inoculated humanized mice at 10 weeks postinfection (wpi). We also quantified the copy number of viral DNA by real-time PCR and revealed that the copy numbers of viral DNA in these 2 mice were comparable (no. 2; 0.38 ± 0.02 copies/cell in spleen and 0.50 ± 0.01 copies/cell in BM; no. 3; 0.77 ± 0.02 copies/cell in spleen and 0.87 ± 0.04 copies/cell in BM). RT-PCR analyses revealed that SRV-4 efficiently replicated in hematogenous and/or lymphoid organs such as BM and spleen of infected mice (Fig. 1b). However, in peripheral blood (PB), proviral DNA was detected only in the mouse no. 10 at 8 wpi (Fig. 2a), and viral RNA was not detected in the plasma of all SRV-4-inoculated mice used in the experiment 1 (Fig. 1b). These results suggest that SRV-4 is able to replicate in humanized mouse model, while is incapable of inducing a high level of viremia.

### Normocytic anemia and hemophagocytosis in SRV-4-infected humanized mice

Since SRV-4 infection in Japanese macaques induced severe thrombocytopenia with anemia^5^,^6^,^7^, we assessed the pathogenic effect of SRV-4 infection in humanized mice. As shown in Figs 1c and 2b, a transient decrease of body weight was occasionally observed in certain SRV-4-infected mice, particularly mouse no. 2. Also, a transient increase of white blood cells was observed in the 2 SRV-4-infected mice (no. 2 and 5; Fig. 1d), and the majority of the expanding leukocytes in the PB of these 2 infected mice was T cells, particularly memory CD8^+^ T cells (Fig. 1l–1t). Furthermore, 3 out of the 8 SRV-4-infected mice (no. 2, 10, and 12) displayed the decreases of red blood cell number (Figs 1e and 2d), hematocrit (Figs 1g and 2f), and hemoglobin (Figs 1h and 2g), suggesting that SRV-4 infection occasionally causes anemia in humanized mice. Further, hematological analyses revealed that the values of mean corpuscular volume (MCV; Figs 1i and 2h), mean corpuscular hemoglobin (MCH; Figs 1j and 2i), and mean corpuscular hemoglobin concentration (MCHC; Figs 1k and 2j), which indicate the quality of red blood cells, in the mice exhibited anemia (Figs 1e and 2d) were comparable to mock-infected mice, strongly suggesting that normocytic anemia occurred in certain SRV-4-infected mice.

Since normocytic anemia is caused by the augmented destruction of red blood cells by phagocytosis^27^, we asked whether phagocytosis is occurred in the infected mouse exhibiting anemia. As shown in Fig. 1u, hematoxylin and eosin (H&E) staining revealed that a large number of macrophage-like phagocytes resided in the hepatic sinusoids and the region close to central vein (CV) in the liver of the infected mouse (no. 2). Also, the infiltrated lymphocytes surround the activated hepatic macrophages (Fig. 1u, upper right), and the degenerated hepatocytes (Fig. 1u, lower right) were observed. Immunostaining revealed that human CD68^+^ macrophages were expanded and the majority of the infiltrating lymphocytes were human CD8^+^ T cells (Fig. 1v). Moreover, Berlin blue staining revealed that these macrophages in the liver of infected mouse contained intracellular hemosiderin, which was derived from engulfed red blood cells (Fig. 1w). Non-specific esterase (NSE) staining revealed that the macrophages engulfing red blood cells were resided in the BM of the SRV-4-infected mice exhibited anemia (Fig. 2k). Interestingly, the hemophagocytosis in BM was particularly observed in the infected mice displayed anemia (Table 2). Taken together, these findings strongly suggest that SRV-4 infection can cause hemophagocytosis leading to normocytic anemia in humanized mice.

### The effect of SRV-4 infection on cytokine production

**Figure 3:**
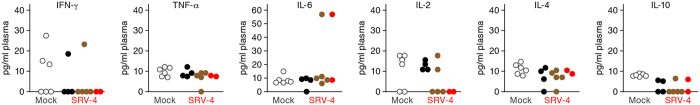
The levels of human cytokines in plasma of SRV-4-infected humanized mice. The amounts of IFN-γ, TNF-α, IL-6, IL-2, IL-4, and IL-10 in the plasma of mock-infected mice (n = 6), SRV-4-inoculated mice without infection (n = 4; black), SRV-4-infected mice without anemia (n = 5; brown), and SRV-4-infected mice exhibited anemia (n = 2; red) at 10 wpi are respectively shown. Horizontal bars indicate the averages of mock-infected mice (n = 6) and SRV-4-infected mice with or without anemia (n = 7), respectively.

We have previously reported that hemophagocytosis and severe anemia can be induced by EBV infection in NOG-hCD34 mice, and that the hemophagocytosis and anemia closely associated with the overproduction of inflammatory cytokines, particularly IFN-γ^23^. To address the possibility that the hemophagocytosis and anemia observed in SRV-4-infected mice is exerted by the augmentation of cytokines, we quantified the amounts of human cytokines in the plasma of infected mice. As shown in Fig. 3, however, the levels of human cytokines including IFN-γ, TNF-α, IL-6, IL-2, IL-4, and IL-10 in the plasma of SRV-4-infected mice were comparable to those in mock-infected mice. These findings suggest that the inflammatory cytokines are not associated with the pathogenic condition in SRV-4-infected humanized mice.

### No adaptation of SRV-4 into humanized mice

**Figure 4:**
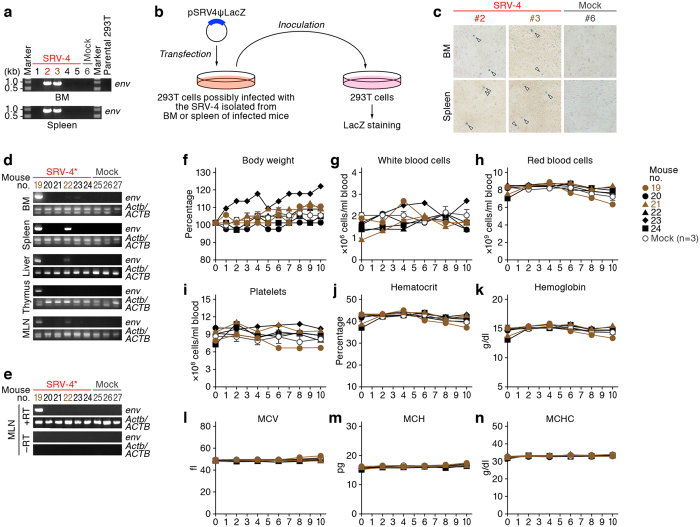
Dynamics of SRV-4* infection in humanized mouse model (experiment 3). (**a-c**) Virus isolation from infected humanized mice. (**a**) The human MNCs isolated from the BM and spleen of SRV-4-inoculated mice were cocultured with 293T cells. DNA was extracted from the cocultured 293T cells and proviral DNA was analyzed by PCR. (**b,c**) LacZ marker rescue assay. The detailed procedure is described in Methods, and representative results of LacZ marker rescue assay are shown in c. Arrowheads indicate the presence of blue foci. (**d,e**) Proviral DNA (**d**) and viral RNA (**e**) were respectively analyzed by PCR and RT-PCR (targeted to *env* region). *Actb*/*ACTB* were used as internal control, and–RT was used as negative control. (**f-n**) Longitudinal analyses on the dynamics of SRV-4* infection. X-axes indicate wpi. (**f**) The body weights were routinely measured and were shown as the ratio to the initial weight. (**g-n**) The numbers of white blood cells (**g**), red blood cells (**h**), platelets (**i**), hematocrit (**j**), hemoglobin concentration (**k**), and the values of MCV (**l**), MCH (**m**), and MCHC (**n**) were measured by hematometry. In f-n, brown, SRV-4-infected mice (i.e., viral DNA was detected; no. 19 and 21); and black, SRV-inoculated mice without viral replication. The data from mock-infected mice (n = 3) were presented as the averages with SEMs. The mouse numbers correspond to those in Table 1. In a, d, and e, gels have been cropped; full uncropped gels are available as Supplementary Figure 3.

Although we did not observe high frequent and pathogenic effect by SRV-4 infection in humanized mice, 8 out of the 13 SRV-4-inoculated mice allowed viral replication, and 3 of the 8 infected mice exhibited normocytic anemia (Figs 1 and 2). These raise a possibility that the SRV-4 replicated and exerted anemia in humanized mice has adapted into human condition and has acquired higher pathogenic potential. To explore this possibility, the human mononuclear cells (MNCs) isolated from the spleen and the BM of 2 humanized mice (no. 2 and 3) were cocultured with 293T cells. The SRV-4 was successfully replicated in *in vitro* coculture with 293T cells (Fig. 4a–c), and sequencing analysis revealed that the SRV-4 isolated from infected humanized mice acquired several mutations in its proviral genome (Table S2). In order to assess the pathogenic potential of the isolated SRV-4, we prepared the SRV-4 solution (SRV-4*), which is isolated from an infected mouse exhibited anemia (no. 2), and inoculated this viral solution (4.8 × 10^5^ TCID_50_) into 6 humanized mice (experiment 3). As shown in Fig. 4d,e, viral DNA and RNA were detected in lines of organs of the 2 SRV-4*-inoculated mice (no. 19 and 22). However, in contrast to the experiments 1 and 2 (Figs 1 and 2), the body weights of SRV-4*-inoculated mice did not change (Fig. 4f), and obvious anemia was not observed in these mice (Figs 4g–n). These findings strongly suggest that the SRV-4, which is replicated in a humanized mouse and induced anemia and hemophagocytosis, is not acquired a higher pathogenic potential in humanized mice.

### Effect of human APOBEC3 proteins against SRV-4 replication

**Figure 5:**
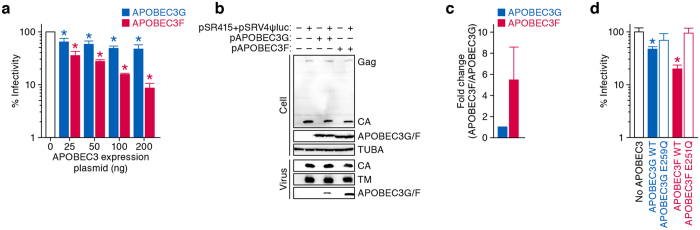
Anti-SRV-4 ability of human APOBEC3 proteins. (**a**) Dose-dependent anti-SRV-4 ability of human APOBEC3F and APOBEC3G. 293T cells were cotransfected with pSR415 (1 μg), pSRV4ψluc (1 μg), and flag-tagged APOBEC3G (blue) or APOBEC3F (red) expression plasmids (25, 50, 100, or 200 ng), and the infectivity of the released virions in the culture supernatant was measured as described in Methods. The infectivity is shown as the percentage of the value of APOBEC3-untransfected cells. (**b,c**) Western blotting. 293T cells were cotransfected with pSR415 (1 μg), pSRV4ψluc (1 μg), and flag-tagged APOBEC3G (blue) or APOBEC3F (red) expression plasmids (200 ng). The expression levels of viral proteins (Gag and CA) and flag-tagged APOBEC3s in the transfected cells, the amount of released virion in the culture supernatant (CA and TM), and the amount of APOBEC3 in the released virion was assessed by Western blotting. The input of cell lysate was standardized to α-tubulin (TUBA), and representative results are shown in b. The amount of APOBEC3s in the released virion was quantified as described in Methods, and the summarized data of the 6 independent experiments are shown in c. (**d**) Deaminase-dependent anti-SRV-4 ability of human APOBEC3F and APOBEC3G. 293T cells were cotransfected with pSR415 (1 μg), pSRV4ψluc (1 μg), and flag-tagged plasmid (200 ng) expressing wild-type (WT) APOBEC3G, APOBEC3G E259Q, WT APOBEC3F, or APOBEC3F E251Q. The infectivity of the released virions in the culture supernatant was measured as described in Methods and is shown as the percentage of the value of APOBEC3-untransfected cells. In a and d, the assays were performed in triplicate. The statistical difference is determined by Student’s *t* test, and statistically significant difference (*P* < 0.05) against no APOBEC3 is indicated by asterisks. Error bars represent SDs. In b, blots have been cropped; full uncropped blots are available as Supplementary Figure 4.

As listed in Table S2, 25 out of the 35 substitution mutations observed (71.4%) were G-to-A mutation. This mutation pattern is reminiscent of the effect caused by human APOBEC3 cytidine deaminases; human APOBEC3 family proteins, particularly APOBEC3F and APOBEC3G, are incorporated into the released particles of HIV-1 and related retroviruses and enzymatically induce G-to-A mutations in the nascent DNA, which results in the disruption viral replication^28^. Since human APOBEC3 proteins potently mutate betaretroviruses such as mouse mammary tumor virus^29^, we asked whether human APOBEC3 proteins affect the infectivity of SRV-4 in *in vitro* cell culture. As shown in Fig. 5a, both APOBEC3F and APOBEC3G suppressed SRV-4 infectivity in dose-dependent manners. Although it is well known that APOBEC3G exhibits more robust anti-HIV-1 activity than APOBEC3F^25^,^30^,^31^, we found that APOBEC3F shows higher anti-SRV-4 activity than APOBEC3G (Fig. 5a). To further address this, we performed Western blotting and found that the incorporation efficacy of APOBEC3F into the released viral particles was 5.5-fold higher than that of APOBEC3G (Fig. 5b,c). Furthermore, we used the catalytically inactive (CI) mutants of APOBEC3G (E259Q) and APOBEC3F (E251Q)^30^ and revealed that these CI mutants did not show anti-SRV-4 effect (Fig. 5d). Taken together, these findings suggest that APOBEC3F exhibits robust anti-SRV-4 activity and that the anti-SRV-4 effect caused by APOBEC3F/G is dependent on their enzymatic activity.

## Discussion

It is known that HIV, the causative agent of acquired immunodeficiency syndrome (AIDS), has emerged in human population by zoonotic transmission of SIVs from non-human primates during the early 20th century^32^,^33^. In addition, it has been recently reported that an avian flu (H5N1) with high pathogenicity and mortality can be transmissible in ferrets, which have a similar immunity to humans, under certain experimental conditions^34^,^35^. These two facts indicate that human population is always exposed to the risk of emerging/re-emerging infection, and that we should keep eyes on the emergence possibility. As mentioned in the Introduction, a highly pathogenic virus in Japanese macaques, SRV-4, has been recently identified in KUPRI^5^,^6^,^7^. Since this virus was isolated by using human cell lines (293T, Raji, and TE671 cells), it seems difficult to exclude the possibility that the virus exhibited a severe illness including hemorrhagic syndrome in the Japanese macaques can be transmitted and cause similar illness in humans. By using humanized mouse model, we demonstrated that 46% (6/13) of the humanized mice inoculated with a high dose of SRV-4 resulted in its infection and that only 23% (3/13) exhibited anemia. Although an infected humanized mouse (no. 12) died exhibiting anemia, the other 2 mice with anemia (no. 2 and 10) recovered without any treatments. Moreover, the adaptation of SRV-4 into humanized mice with higher pathogenicity was not observed. Altogether, these findings strongly suggest that the zoonotic transmission of pathogenic SRV-4 into human population may not occur.

In a previous study, EBV infection in our humanized mice displayed severe illness accompanied by anemia and hemophagocytosis^23^. In that case, 28 humanized mice were inoculated with EBV and 71% (20/28) of the inoculated mice presented severe anemia and resulted in fatal outcome within 10 weeks^23^. EBV-infected mice exhibited hemophagocytosis in BM and liver, erythropenia, thrombocytopenia, expansion of activated CD8^+^ T cells and their infiltration into organs such as liver, and IFN-γ hypercytokinemia^23^, suggesting that our humanized mouse model has the potential to reproduce anemia caused by virus infection. In this study, only 3 out of the 13 SRV-4-infected mice displayed normocytic anemia, the infiltration of CD8^+^ T cells into liver, and the expansion of phagocytosing macrophages in liver and BM. These are reminiscent of the observations in EBV-infected humanized mice^23^. However, the SRV-4-infected humanized mice exhibiting disorders did not show high and prolonged viremia, which have been observed in the moribund Japanese monkeys infected with SRV-4^5^,^6^,^7^, Moreover, viral RNA was hardly detected in the plasma of all SRV-4-inoculated mice, suggesting that SRV-4 cannot induce viremia and is less replicative in humanized mouse model when compared to Japanese macaques. Furthermore, IFN-γ hypercytokinemia, a hallmark of EBV-infected humanized mice with fatal anemia^23^, was not observed in SRV-4-infected humanized mice. Therefore, these findings suggest that SRV-4 is less replicative and rarely induces severe hemophagocytosis leading to anemia in our humanized mice.

In humans, hemophagocytosis is often accompanied by cytokine storm^36^, but our data do not support this scenario in our model for SRV-4 infection (Fig. 3). Regarding the trigger of accelerated hemophagocytosis in SRV-4-infected mice, at least 2 possibilities can be raised; the involvement of unanalyzed inflammatory cytokines and the direct infection of SRV-4 into macrophages/phagocytes. Related to the latter possibility, we revealed that human CD68+ macrophages/phagocytes are highly activated and phagocytosing in the liver of the SRV-4-infected mice with exhibiting anemia (Fig. 1u–w). Importantly, betaretroviruses including SRV-4 are able to replicate only in proliferating/dividing cells^37^. Therefore, it is plausible that SRV-4 can replicate in the activated phagocytes/macrophages, which may accelerate phagocytosis.

Based on the observations of the moribund Japanese macaques infected with SRV-4 exhibited acute anemia with thrombocytopenia, it is implied that the SRV-4 infection in BM is attributed to fatal anemia in Japanese macaques^5^,^6^,^7^. In this study, SRV-4 infection was observed in the BM of the 3 humanized mice with anemia (no. 2, 10, and 12). Since the NOG mice without human HSC xenotransplantation were not infected with SRV-4 (data not shown), murine cells and organs are not susceptible to SRV-4 infection, and the human cells reconstructed in the BM of human HSC-transplanted humanized mice were infected with SRV-4.

It is noteworthy that humanized mouse models do not possess the potential to induce efficient acquired immune responses against pathogens^26^. Even such condition, SRV-4 at a higher dose (more than 10^5^ TCID_50_) rarely induced severe illness, further suggesting that SRV-4 is neither efficiently transmitted into human population as zoonosis nor causes severe illness in infected persons. Importantly, it has been recently reported that SRV-4 infection can be efficiently inhibited by certain anti-HIV-1 drugs^5^. Therefore, the penetration and spread of SRV-4 in human population will be controlled by these drugs if the persons are occupationally exposed to SRV-4 from the Japanese macaques exhibiting severe illness.

Interestingly, the SRV-4 isolated from infected humanized mice exhibited APOBEC3-associated mutation signature (Table S2). We also revealed that APOBEC3F more robustly affected SRV-4 infectivity *in vitro* (Fig. 5). These observations suggest that the APOBEC3F endogenously expressed in human cells closely associated with the G-to-A mutations detected in the infected humanized mice. Moreover, we have recently reported that APOBEC3F-mediated G-to-A mutations potently enhance the diversification and evolution of HIV *in vitro*^30^ and in our humanized mouse model^25^. Because SRV-4 is more susceptible to APOBEC3F than APOBEC3G (Fig. 5), the APOBEC3F-mediated mutation may drive the evolution and diversification of SRV-4.

In conclusion, our findings demonstrate that the zoonotic SRV-4 infection from Japanese macaques to humans would rarely occur. Moreover, this study also provides us a novel issue that humanized mouse model can be used as the initial animal model to screen the emerging/re-emerging pathogens, which exert the possibility to cause zoonotic infection into human population.

## Methods

### Ethics statement

All procedures including animal studies were conducted following the guidelines for the Care and Use of Laboratory Animals of the Ministry of Education, Culture, Sports, Science and Technology, Japan. The authors received approval from the Institutional Animal Care and Use Committees (IACUC)/ethics committee of Kyoto University institutional review board (protocol number D13-25). All protocols involving human subjects were reviewed and approved by the Kyoto University institutional review board. Informed written consent from human subjects was obtained in this study.

### Humanized mice

NOD.Cg-*Prkdc*^*scid*^ *Il2rg*^*tm1Sug*^/Jic mice (NOG mice)^38^ were obtained from the Central Institute for Experimental Animals (Kanagawa, Japan). The mice were handled in accordance with the Regulation on Animal Experimentation at Kyoto University. Human HSCs were isolated from human fetal liver as described previously^39^. NOG-hCD34 humanized mouse was constructed as previously described^19^,^20^,^21^,^23^,^24^. Briefly, 27 newborn (aged 0 to 2 days) NOG mice from 6 litters were irradiated with X-ray (10 cGy per mouse) by an RX-650 X-ray cabinet system (Faxitron X-ray Corporation) and were then intrahepatically injected with the obtained human HSCs (10 × 10^4^ to 17 × 10^4^ cells). A list of the humanized mice used in this study is summarized in Table 1. Although an SRV-4-inoculatd mouse (no. 12) exhibited moribund condition (see below for detail) with anemia and acutely died, the other 18 mice used in this study were anesthetized and sacrificed at 10 wpi as previously described^23^.

### Cell culture

293T cells, TE671 cells^18^, and TELCeB6 cells^40^ were maintained in Dulbecco’s modified Eagle medium (DMEM) containing 10% fetal calf serum (FCS) and antibiotics as previously described^8^,^20^,^21^,^41^,^42^,^43^.

### Virus preparation, titration, and infection. (i) Experiment 1

Virus solution was prepared as previously described^8^. Briefly, the culture supernatant of the TE671 cells persistently infected with a molecular clone of SRV-4 (pSR415)^6^ were harvested, filtrated with 0.45-μm-pore-size filter (Millipore), and used as virus solution. Because the infectivity of virus solution severely decreases by freeze-and-thaw^6^, 1.5 ml of the virus solution was immediately inoculated into 6 humanized mice intraperitoneally. The infectivity of virus solution was determined as TCID_50_ by LacZ marker rescue assay using TELCeB6 cells as previously described^6^. In experiment 1, the infectivity of the virus solution used was 3.2 × 10^5^ TCID_50_/ml, and the amount of viral RNA of the virus solution used was 3.8 × 108 copies/ml. **(ii) Experiment 2.** Virus solution (5.6 × 10^5^ TCID_50_/ml; 4.4 × 10^8^ copies/ml) was prepared independently of the experiment 1, and 1.5 ml of the virus solution was intraperitoneally inoculated into 7 humanized mice. **(iii) Experiment 3.** The virus solution isolated from mouse no. 2 (SRV-4*) was prepared as described below (see “Virus isolation from infected mice” section), and 1.5 ml of the isolated virus solution (3.2 × 10^5^ TCID_50_/ml; 2.0 × 10^8^ copies/ml) was intraperitoneally inoculated into 6 humanized mice. In all three independent experiments, DMEM was used in mock infections.

### PB collection, isolation of mononuclear cells from organs, flow cytometry, and hematometry

PB and plasma were routinely collected as previously described^19^,^20^,^21^,^23^,^24^. Spleen, MLN, thymus, and liver was crushed, rubbed, and suspended, and BM was obtained from the dissected thighbones by flushing the interior as previously described^23^. Human MNCs in the spleen and the BM were isolated as previously described^23^. Flow cytometry and hematometry were performed as previously described^19^,^20^,^21^,^23^,^24^. Flow cytometry was performed with FACSCanto II and FACSCalibur (BD Biosciences), and the obtained data were analyzed with CellQuest software (BD Biosciences) and FlowJo software (Tree Star, Inc.). Hematometry was performed with Celltac α MEK-6450 (Nihon Kohden, Co.).

### Histological analyses and immunostaining

The liver section was prepared as previously described^23^. H&E staining, Berlin blue staining, and NSE staining were respectively performed with BZ-8000 microscope (Keyence) as previously described^23^. Immunostaining was performed with Leica TCS SP2 AOBS confocal laser microscope (Leica Microsystems) as previously described^19^,^21^,^23^ by using the following antibodies: rabbit anti-human CD8 monoclonal antibody (Lab Vision) and mouse anti-human CD68 monoclonal antibody (BD Biosciences). Nuclei were stained with Hoechst33342 (Life Technologies).

### PCR, RT-PCR, and real-time PCR

DNA and RNA were respectively extracted by using DNeasy kit (Qiagen) and RNeasy kit (Qiagen) according to the manufacture’s protocol. To detect SRV-4 DNA (*env* region) and *ACTB/Actb* (human and murine β-actin genes), PCR was performed by using *ExTaq* DNA polymerase (Takara) and the primers listed in Table S1. Reverse transcription was performed by using SuperScript III reverse transcriptase (Life Technologies) according to the manufacture’s protocol. RT-PCR was performed by using PrimeSTAR GXL DNA polymerase (Takara) and the primers described above. Real-time PCR was performed by using Power SYBR green PCR master mix (Applied Biosystems) and the primers listed in Table S1 as previously described^6^.

### Plasmid construction

pSRV4ψLacZ (a LacZ-expressing reporter plasmid with a SRV-4 packaging signal) and pSRV4ψluc (a luciferase-expressing reporter plasmid with a SRV-4 packaging signal) were respectively constructed by replacing the nucleotides from 801th to 7594th of pSR415 (the transcription initiation site was defined as position +1) with SV40 promoter-*nls*LacZ and SV40 promoter-luciferase using In-Fusion HD Cloning kit (Clontech)^6^,^8^.

### Virus isolation from infected mice

To isolate the SRV-4 replicating in humanized mice, 5 × 10^5^ of human MNCs isolated from the BM and the spleen of 2 SRV-4-infected mice (no. 2 and 3) were cocultured with 5 × 10^5^ of 293T cells. The cocultured 293T cells were maintained for 7 days, and the cells stored at –80°C for DNA extraction, PCR, and sequencing analyses. For sequencing analysis, two portions of SRV-4 provirus (5′ portion, 1–2036; 3′ portion, 1811–8126) were amplified by PCR using the primers listed in Table S1. Sequencing was performed by using the primers listed in Table S1. The culture supernatant at day 7 was centrifuged, filtrated with 0.45-μm-pore-size filter (Millipore), and further inoculated into fresh 293T cells. The inoculated 293T cells were maintained by passaging by 3 days for 2-3 weeks. To determine the persistent infection of the isolated SRV-4 from inoculated mice (no. 2 and 3) in the 293T cells, SRV-4 LacZ marker rescue assay was performed as previously described^6^. Briefly, pSRV4ψLacZ was transfected into the 293T cells by using Lipofectamine 2000 (Life technologies) according to the manufacture’s protocol. At 48 hours posttransfection, the culture supernatant was harvested, filtrated with 0.45-μm-pore-size filter (Millipore), and further inoculated into fresh 293T cells. Then, LacZ staining was performed at 48 hour postinoculation (see also Fig. 4b). The SRV-4 isolated from the BM of the humanized mouse no. 2 (SRV-4*) was used for the experiment 3 (see above).

### Cytokine quantification

The amounts of human cytokines including interferon (IFN)-γ, tumor necrosis factor (TNF)-α, interleukin (IL)-6, IL-2, IL-4, and IL-10 in plasma were quantified by using BD Cytometric Bead Array (CBA) Human Th1/Th2 Cytokine Kit II (BD Biosciences) according to the manufacture’s protocol^23^.

### Transfection, reporter assay, and Western blotting

To evaluate the anti-SRV-4 effect of human APOBEC3, pSR415 (1 μg) and pSRV4ψluc (1 μg) were cotransfected with pCMV-flag-APOBEC3F or pCMV-flag-APOBEC3G and their CI mutants (APOBEC3F E251Q and APOBEC3G E259Q^25^,^30^ into 293T cells by using Lipofectamine 2000 (Life Technologies) as previously described^25^,^30^. At 48 hours posttransfection, the culture supernatant was harvested, filtrated with 0.45-μm-pore-size filter (Millipore), and further inoculated into fresh 293T cells. At 48 hours postinoculation, the luciferase activity was measured as previously described^42^ and determined as viral infectivity. The expression levels of APOBEC3 and viral proteins were measured by Western blotting as previously described^25^,^30^,^42^. Briefly, mouse anti-flag monoclonal antibody (M2; Sigma), mouse anti-α-tubulin (TUBA) monoclonal antibody (DM1A; Sigma), rabbit anti-SRV-4 CA polyclonal antibody^8^, rabbit anti-SRV-4 TM polyclonal antibody^8^ were used. To quantify the amount of released virion and APOBEC3 proteins in virion, the virus solution was ultracentrifuged and used for Western blotting as previously described^25^,^30^,^42^. The level of APOBEC3 proteins in virion was quantified by using ImageJ software (http://rsbweb.nih.gov/ij/) as previously described^21^.

### Statistical analyses

Data were expressed as averages with standard errors of the means (SEMs) or standard deviations (SDs). Statistical differences were determined by Student’s *t* test.

## Additional Information

**How to cite this article**: Sato, K. *et al*. Experimental evaluation of the zoonotic infection potency of simian retrovirus type 4 using humanized mouse model. *Sci. Rep*. **5**, 14040; doi: 10.1038/srep14040 (2015).

## Supplementary Material

Supplementary Information

## Figures and Tables

**Table 1:** Humanized mice used in this study.^a^

Mouse no.^b^	Recipient mice		Transplanted hHSCs		Inoculated virus^e^	Dose (x10^5^ TCID_50_)	Inoculation age (weeks old)
	Lot no.^c^	Sex	Donor lot^d^	Cell number			
1	131	M	a	120,000	SRV-4	4.8	11
2	131	M	a	120,000	SRV-4	4.8	11
3	132	F	a	120,000	SRV-4	4.8	11
4	132	F	a	120,000	SRV-4	4.8	11
5	132	F	a	120,000	SRV-4	4.8	11
6	132	F	a	120,000	–		11
7	132	M	a	120,000	–		11
8	132	M	a	120,000	–		11
9	143	F	b	170,000	SRV-4	8.4	19
10	143	F	b	170,000	SRV-4	8.4	19
11	144	F	c	100,000	SRV-4	8.4	18
12	144	F	c	100,000	SRV-4	8.4	18
13	144	F	c	100,000	SRV-4	8.4	18
14	144	M	c	100,000	SRV-4	8.4	18
15	144	M	c	100,000	SRV-4	8.4	18
16	143	F	b	170,000	–		19
17	144	F	c	100,000	–		18
18	144	M	c	100,000	–		18
19	138	F	c	110,000	SRV-4*	4.8	14
20	138	M	c	110,000	SRV-4*	4.8	14
21	141	M	c	110,000	SRV-4*	4.8	12
22	141	M	c	110,000	SRV-4*	4.8	12
23	141	M	c	110,000	SRV-4*	4.8	12
24	141	M	c	110,000	SRV-4*	4.8	12
25	138	F	c	110,000	–		14
26	138	F	c	110,000	–		14
27	141	M	c	110,000	–		12

^a^hHSCs, human CD34^+^ hematopoietic stem cells; M, male; F, female.

^b^The mouse numbers correspond with those in the Figures.

^c^Six lots of new born NOG mice were used for the recipient.

^d^NOG-hCD34 mice were reconstituted with one of three donors.

^e^SRV-4*, the virus isolated from the BM of an SRV-4-infected mouse (no. 2).

**Table 2:** Summary of the observations in the experiment 2.

Mouse no.^a^	Viral DNA^b^		Anemia^c^	NSE staining^d^		Notes
	BM	Spleen		Macrophages^e^	Phagocytosis^f^	
9	–	–	–	+	+/−	
10	+	+	+	++	+	Exhibited anemia and recovered.
11	+/−	+/−	–	+	–	
12	+	+	+	*NA*	*NA*	Exhibited anemia and suddenly died.
13	+	+	–	+	+/−	
14	+	+	–	+	–	
15	+	+	–	+	–	
16	–	–	–	–	–	
17	–	–	–	+/−	–	
18	–	–	–	–	–	

*NA*, not applicable because the BM fluid specimen was not obtained.

^a^The mouse numbers correspond with thos in Table 1 and Fig. 2.

^b^The results of PCR analyses (Fig. 2a) were summarized.

^c^The results of hematological analyses (Figs 2d,f,g) were summarized.

^d^The observation on NSE staining was assessed by the two hematologists (J.Y. and M.M.).

^e^macrophages were : ++, frequently observed ; +, observed ; +/−, less observed ; −, not observed.

^f^phagocytosing macrophages were : +, observed ; +/−, less observed ; −, not observed.
