# Supplementary material for: Experimental evaluation of the zoonotic infection potency of simian retrovirus type 4 using humanized mouse model
Source: Sci Rep. 2015 Sep 14;5:14040. doi: 10.1038/srep14040 (PMC4568461; doi:10.1038/srep14040)
Supplement: Supplementary Information [file srep14040-s1.pdf]

Experimental evaluation of the zoonotic infection potency of simian retrovirus type 4 using humanized mouse model

Kei Sato, Tomoko Kobayashi, Naoko Misawa, Rokusuke Yoshikawa, Junko S. Takeuchi, Tomoyuki Miura, Munehiro Okamoto, Jun-ichirou Yasunaga, Masao Matsuoka, Mamoru Ito, Takayuki Miyazawa and Yoshio Koyanagi

Supplementary information:

Supplementary figures 1-3: original (uncropped) gels
Supplementary figure 4: original (uncropped) blots
Supplementary table 1: primers used in this study
Supplementary table 2: mutations in the SRV-4 isolated from the BM of infected humanized mice

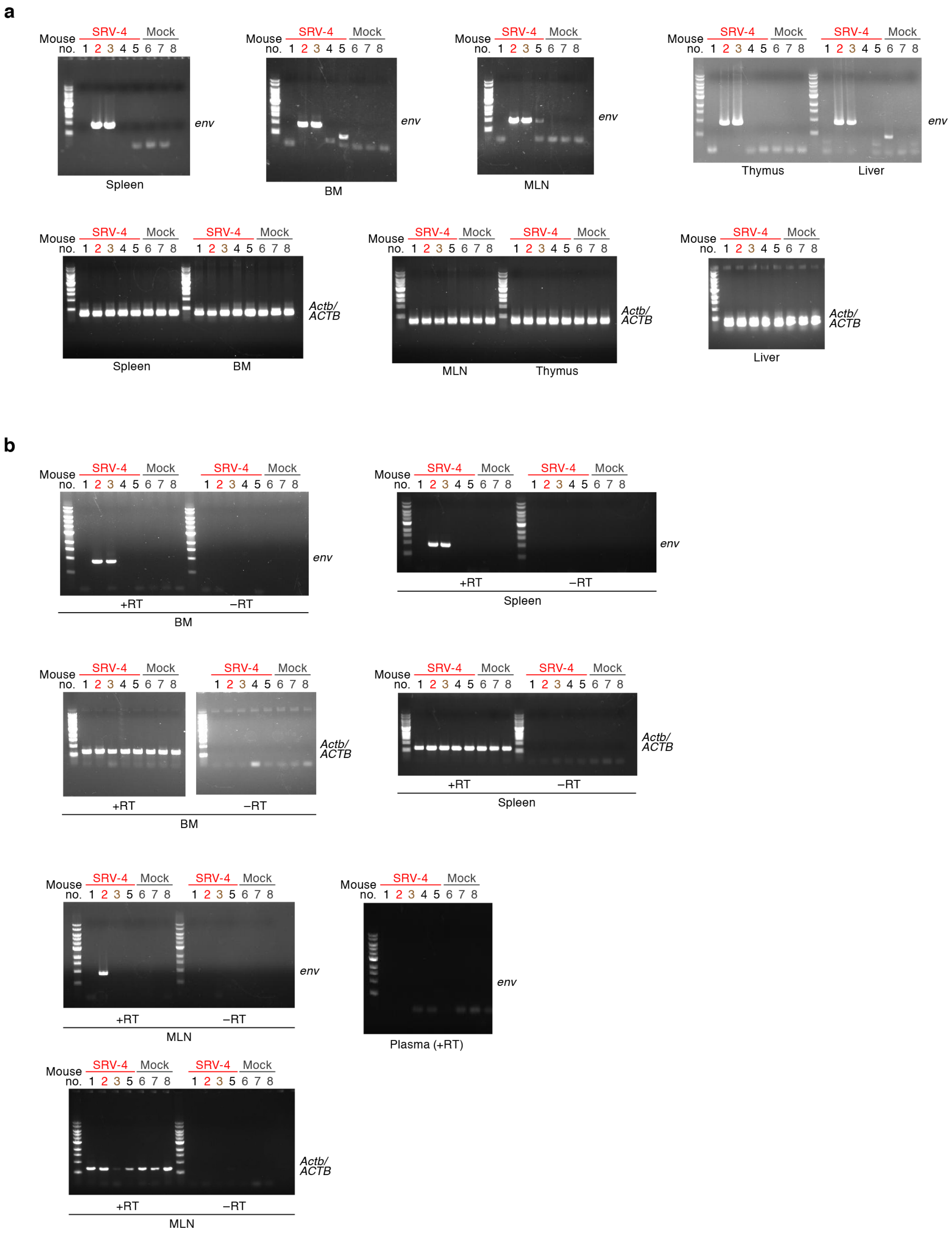

Supplementary figure 1. Original (uncropped) gels of Figures 1a and 1b.

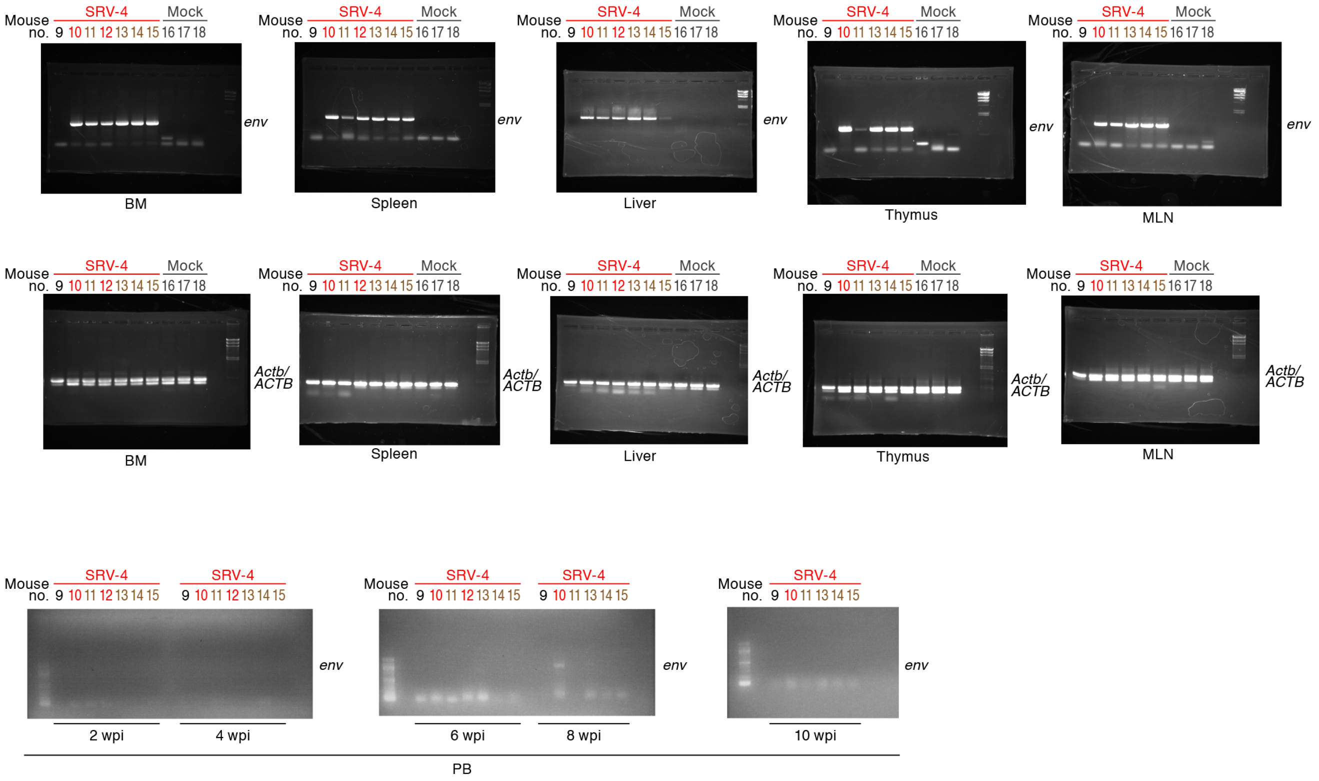

Supplementary figure 2. Original (uncropped) gels of Figure 2a.

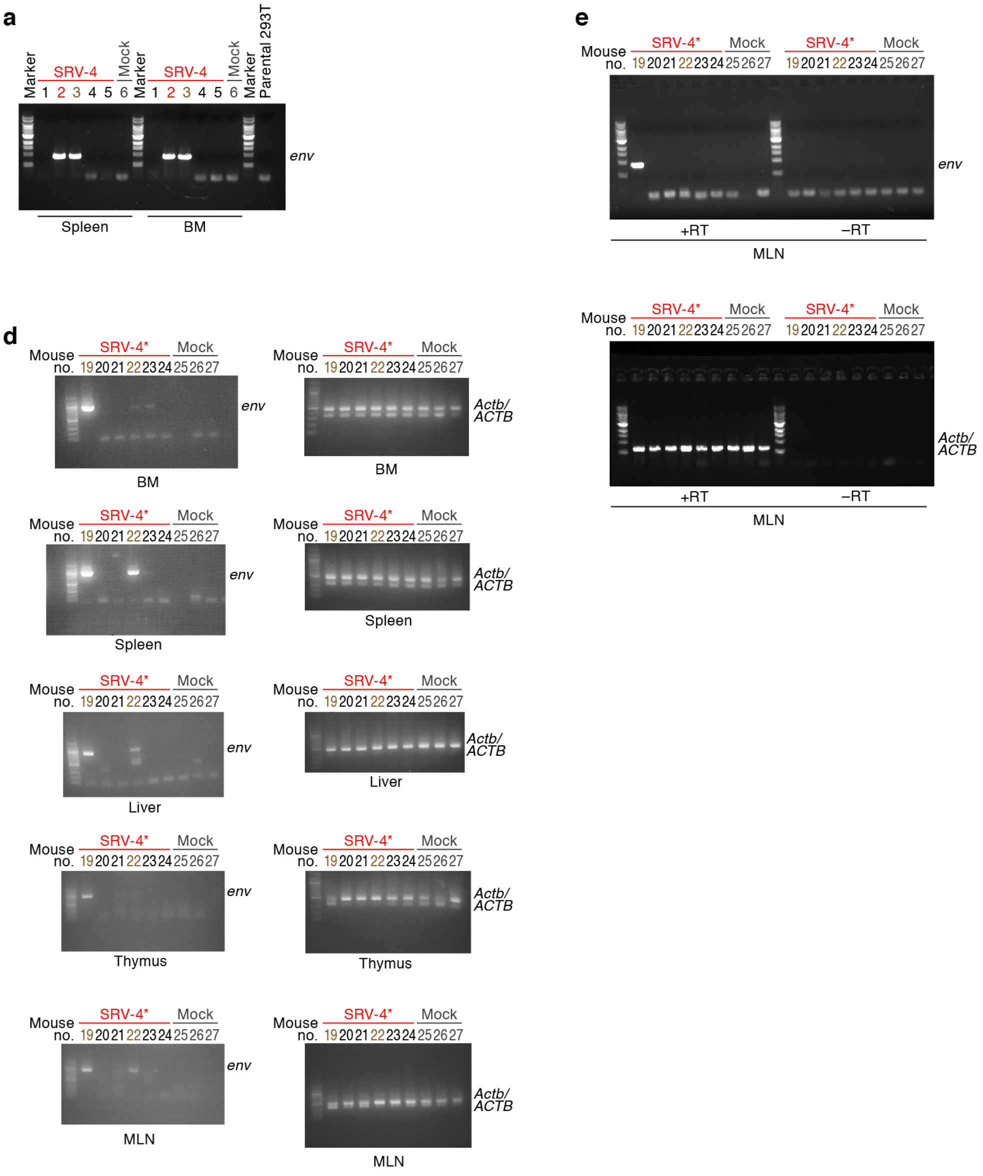

Supplementary figure 3. Original (uncropped) gels of Figures 4a, 4d, and 4e.

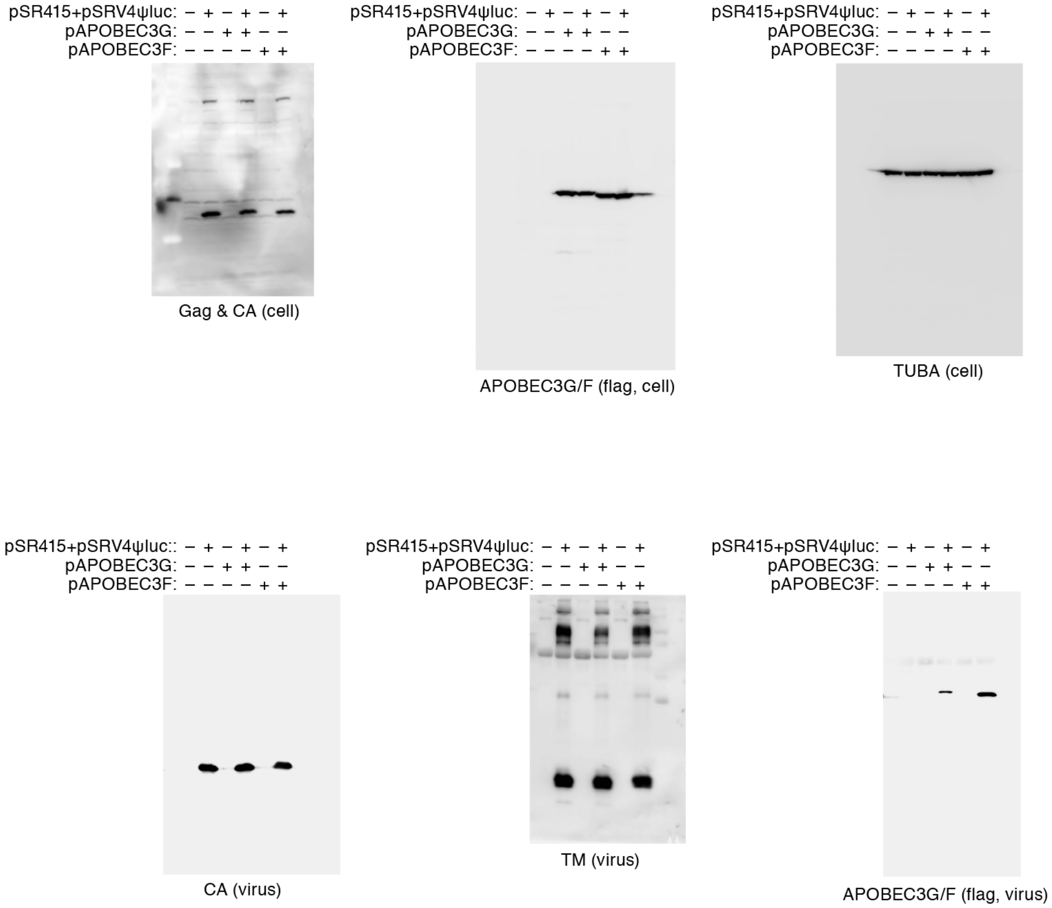

Supplementary figure 4. Original (uncropped) blots of Figure 5b.

Table S1. Primers used in this study.

Name	Sequence (5'-to-3')	Target	Usage
SRV-4 *env* forward (6034-6057)	TTCTCACACTGCATACCAACCTAC	SRV-4	PCR/RT-PCR
SRV-4 *env* reverse (6933-6912)	AGGGTGACAAGTACACACCTTC	SRV-4	PCR/RT-PCR
SRV4 *env* realtimeF2	TGCGAGTGCAAAGGAGGATAT	SRV-4	Real-time PCR
SRV4 *env* realtimeR2	CGCAAGAAATGGCTGCAA	SRV-4	Real-time PCR
ACTB/Actb forward	TGGAATCCTGTGGCATCCATGAAAC	*ACTB/Actb*	PCR/RT-PCR
ACTB/Actb reverse	TAAAACGCAGCTCAGTAACAGTCCG	*ACTB/Actb*	PCR/RT-PCR
SRV4-5' forward (1-20)	TGTCCGGAGCCGTGCGGCCC	SRV-4	Provirus amplification
SRV4-5' reverse (2036-2013)	CCGAACAGAGACGGATATATCCAG	SRV-4	Provirus amplification
SRV4-3' forward (1811-1830)	GACCCAGGAGCTTCGCTCAC	SRV-4	Provirus amplification
SRV4-3' reverse (8126-8105)	TGTCCCGTCCCGCGGGATCAAC	SRV-4	Provirus amplification
SRV4-5seq1 (505-524)	CCAACATGAAAAGTATATAG	SRV-4	Sequencing
SRV4-5seq2 (996-1014)	ATCATAACCCTGACCCTTC	SRV-4	Sequencing
SRV4-5seq3 (1501-1520)	TACTGCTCCGTATACCTTAG	SRV-4	Sequencing
SRV4-3seq1 (2311-2330)	GGGAAACGGACTGAGGGGCC	SRV-4	Sequencing
SRV4-3seq2 (2810-2829)	TATGGTTAGATGGCAAACCC	SRV-4	Sequencing
SRV4-3seq3 (3321-3340)	ACAAAGGAAAAACTTACAGC	SRV-4	Sequencing
SRV4-3seq4 (3851-3870)	AAAGTTAGTTATAGCTGGAC	SRV-4	Sequencing
SRV4-3seq5 (4411-4430)	CCAAATCTCAAATAGATTGG	SRV-4	Sequencing
SRV4-3seq6 (4961-4979)	AGCCTTTAATCTCAATACG	SRV-4	Sequencing
SRV4-3seq7 (5501-5520)	AGAATGGTACCCTACAAAGG	SRV-4	Sequencing
SRV4-3seq8 (6031-6050)	TGGTTCTCACACTGCATACC	SRV-4	Sequencing
SRV4-3seq9 (6611-6630)	CCCGTACCCCTTGCCTTACC	SRV-4	Sequencing
SRV4-3seq10 (7173-7192)	AAGACCAGGTAGATTCTTTG	SRV-4	Sequencing
SRV4-3seq11 (7714-7731)	ATAAGAAATGCATCACTC	SRV-4	Sequencing

Table S2. Mutations in the SRV-4 isolated from the BM of infected humanized mice.

Mouse no.	Clone	Substitutions											Other mutations
No. 2	1	**G3804A**	**G5950A**										
		(Gly→Arg)	(syn.)										
	2	**A3829T**	**G3849A**	**T4916A**	**C5369T**								Deletion (1831-1856)
		(Gln→Leu)	(Glu→Lys)	(syn.)	(syn.)								
	3	**A4188C**	**G5733A**										
		(Ile→Leu)	(Ala→Thr)										
No. 3	1	**T308C**	**T1456C**	**G1833A**	**G2743A**	**G2752A**	**G3494A**	**G3510A**	**G3524A**	**G5547A**	**G7368A**	**A7493C**	
		NA	(syn.)	(syn.)	(Gly→Arg)	(Gly→Arg)	(Met→Ile)	(Gly→Arg)	(syn.)	(Ala→Thr)	*NA*	*NA*	
	2	**T308C**	**G2992A**	**G5547A**	**G7368A**	**A7493C**	**T8096C**						
		NA	(Asn→Asp)	(Ala→Thr)	(Asp→Gly)	(Ile→Leu)	*NA*						
	3	**G1833A**	**G2035A**	**G2086A**	**G2253A**	**G2287A**	**G2478A**	**G2752A**	**G4881A**	**G5415A**	**G5576A**		Insertion (5630A)
		(Gly→Glu)	(syn.)	(Gly→Arg)	(Trp→stop)	(Gag: syn.) (Prt: Gly→Arg)	(Gly→Arg)	(Gly→Arg)	(Gly→Arg)	(Gly→Arg)	(syn.)		

syn., synonymous ; *NA,* no applicable because of the mutation on LTR region.
